# Recovery of Phytochemicals via Electromagnetic Irradiation (Microwave-Assisted-Extraction): Betalain and Phenolic Compounds in Perspective

**DOI:** 10.3390/foods9070918

**Published:** 2020-07-12

**Authors:** Moh Moh Zin, Chukwuka Bethel Anucha, Szilvia Bánvölgyi

**Affiliations:** 1 Department of Food Engineering, Szent István University, Ménesi út 44, 1118 Budapest, Hungary; Banvolgyi.Szilvia@etk.szie.hu; 2 Department of Chemistry, Karadeniz Technical University, 61080 Trabzon, Turkey; chikadoog@ktu.edu.tr

**Keywords:** betalains, phenolic compounds, antioxidants, microwave irradiation, emerging technology, phytochemicals

## Abstract

Food colorants processed via agro-industrial wastes are in demand as food waste management becomes vital not only for its health benefits but also for cost reduction through waste valorization. Huge efforts have been made to recover valuable components from food wastes and applied in various fields to prove their versatility rather than for feed ruminant usage only. Betalains and phenolics, antioxidant-rich compounds responsible for host color and so commonly used as natural colorants in food and cosmetic industries, are copiously present in several kinds of fruits and vegetables as well as their wastes. Technological innovation has brought extensive convenient ways of bioactive compounds extraction with many advantages like less use of solvents and energy in a short period of processing time in comparison with the classical solid–liquid extraction methods. Emerging technologies, particularly microwave irradiation, have been amenable to electromagnetic technology for decades. Practically, they have been deployed for functional and supplement food production. In this review, the feasibility of dielectric heating (microwave irradiation) in the extraction of betalain and phenolic compounds mostly from fruit and vegetable wastes was discussed.

## 1. Introduction

Global interest for natural food colors is on the rise, especially in Asian countries. Historically, food ingredients and spices like yellow turmeric and red pepper powders are easily prone to food adulteration [1]. They play very essential roles in traditional cuisines and are valued for their therapeutic properties irrespective of their color. Current innovation and developmental trend in food processing has led to a paradigm interest in food additives and bioactive compounds with particular focus on their nutritional and organoleptic values.

Among the bioactive compounds, betalains and phenolics are the most auspicious with plenty of health benefits, making them easily compatible with food fortification and supplementation. The discovery of betanin, betanidin, and indicaxanthin derivatives following their degradation led to the assumption of their unique nature together with betaxanthin (formerly known as flavonoid) and betacyanin (regarded as nitrogenous anthocyanin) [2]. This led to them being grouped as betalains in 1968 [3]. Among the acknowledged sources of betalains, i.e., beetroot, swiss chard, dragon fruit, and prickly pear [3,4,5,6], beetroot has gained the most attention since, apart from the whole tuber, even their waste parts such as stalk [7], peel [8,9], and pomace [10,11] are rich in betalains. The ratio of color contents is decisively influenced by specie source, genetic, cultivar, or the environment of cultivation throughout growing, and the stages of harvesting [4,12,13]. Practically, one kilogram of fresh beetroot contains 300–600 mg of betacyanin and 320–420 mg of betaxanthin [14]; compared to the flesh, betalain concentration ranges from 40 to 70% higher in the peel and it also depends on genotypes [15].

As reducing agents, phenolic compounds can inhibit oxidation reaction catalyzed by enzymes and can stabilize lipid peroxidases [16]. Over 10,000 phenolic compounds exist in several kinds of fruits and vegetables, cereals, tea leaves, coffee beans, and their accumulation in the whole parts of the plant including root, stem, bark, flower, and leaf have been noticed [17]. Their extraction from various sources has been boosted by emerging technologies these days, particularly microwave radiation. Some of them are twenty cultivars of tomatoes [18], six plant species [19], four types of spices [16], four types of algae (brown macroalgae species) [20], and the rest are discussed in the next section with their proper process optimization. Balasundram and coworkers [21] reviewed phenolic compound structures found in by-products of fruit and vegetable processing. Extraction of phenolic compounds from these food products, however, remains a developing field as different techniques are continually being explored.

Compared to conventional extraction techniques, non-conventional methods are more pronounced for better quality and quantity of desired products due to advantages such as less use of solvent and lower exposure time to high temperature [22]. Specifically, major advantages of microwave treatments are the reduction in operation time and solvent consumption (critically possibility of solvent-free processing), less energy consumption as the rays are delivered directly to the matrix, as well as their ease of handling and processing [23]. As reported by Cardoso-Ugarte and coworkers [24], the same amount of betalain concentration can be achieved by microwave irradiation ten times faster than the conventional ways with betalain yield scaled up to double. However, with dielectric heating, acceleration in the chemical reaction of target compounds such as epimerization, oxidation, and polarization during microwave processing should be considered [14]. The aim of this review was to point out the versatility of microwave irradiation in the recovery of bioactive compounds focusing on phenolics and betalains from various kinds of fruits and vegetables for their improved practical application in food and beverages, pharmaceuticals, cosmetics, and other related sectors.

## 2. Food Color Compounds

Functional additives are compulsory in food processing in order to improve or maintain the existing properties of food during processing [25]. Bio-colorants have been explored from plants (flower, root, stalk, seed, fruit, peel, leaf, pomace, rhizome, and stigma), insect (cochineal), algae, bacteria, and fungi [25,26,27] and their existence is essential for pollination and seed disposal as insects and birds are attracted by their hues [28]. Carotenoids, phenolics, alkaloids, nitrogen-containing compounds, and organosulfur compounds are major classes of bioactive compounds. Among them, carotenoids (E160, E161, E164), chlorophylls (E140, E141), flavonoids (anthocyanin (E163)), and betalains (E162) are commonly used as natural colorants in food and cosmetic industries (Table 1) [3,25,28]. Since these compounds have characteristic absorption in the visible spectrum, it is possible to quantify them through UV-visible spectrophotometer within 380–700 nm wavelength range following Beer–Lambert’s law.

- Carotenoids, 40 carbon atoms possessing terpenoids, are derived from the condensation of geranylgeranyl-PP molecules [29]. They are lipid-soluble, basically found in cyanobacteria, algae, plants, some fungi and bacteria, and are produced intracellularly by bioproduction of microorganisms [29,30]. Based on their chemical structure, they can be classified as hydrocarbon carotenoids and xanthophylls, and their extraction can easily be performed with nonpolar solvents [29].
- Flavonoids are water-soluble compounds, consisting of 15 carbon atoms (C6-C3-C6) and belong to the class of phenylpropanoids [21,31,32]. Flavonoids furnish intense color, texture, and taste in fruits and flowers, stretching to a wide range of fruit and vegetable parts mostly leaves, flowers, and skin of the fruits [17,27]. The color variety and classification differ according to the structural groups such as hydroxyl, methyl, glucosyl, and acyl [32]. Anthocyanins (glycosylated and acylated) are groups of flavonoids derived from phenylalanine conferring coloration from pale yellow to blue with respect to pH changes [32]. Anthocyanins possess several therapeutic properties as they strongly exhibit free radical scavenging capacity [17]. They are abundantly found in berries, blackcurrant, and other purple color giving fruits and vegetables with host taste attributes [17,28].
- Both anthocyanin and betalain (betacyanin) have UV protectable ability for host plant tissues [28]. Betalains have a wider range of pH (3–7) with yellow to red coloration within that range though less stable to temperature and light exposure as compared to anthocyanin [33]. Slavov and coworkers [34] investigated the color pattern resulting from a mixture of betalain and anthocyanin-rich fruit juices accompanying their functional properties as their coexistence has never been explored.

### 2.1. Betalains

**Figure 1:**
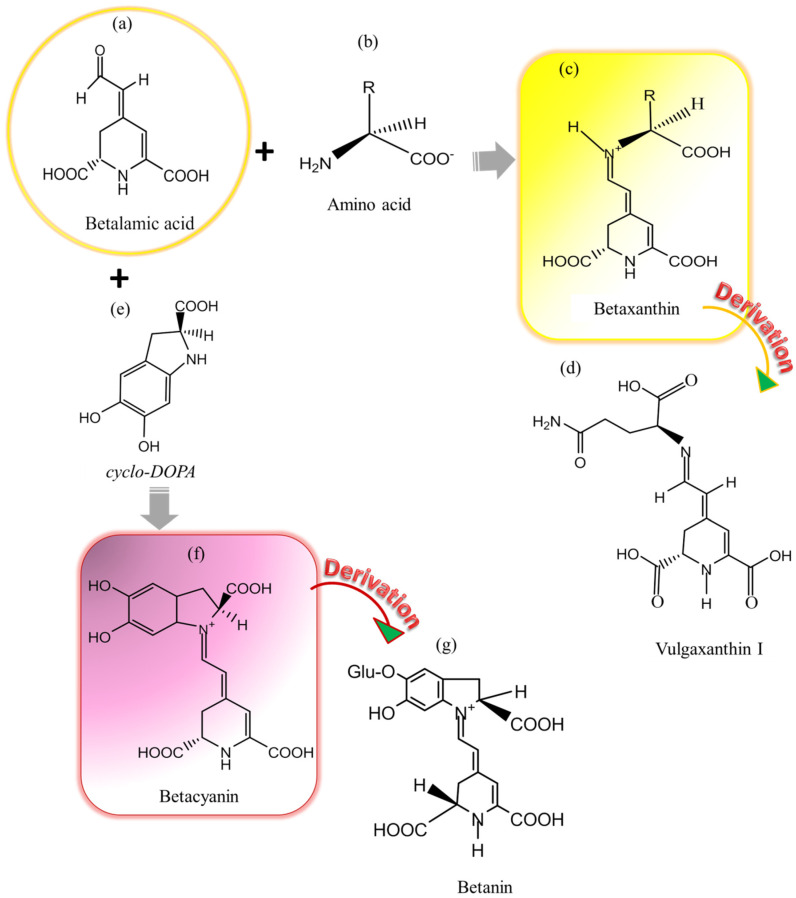
Derivation of betacyanin and betaxanthin color compounds from betalamic acid: (**a**) Betalamic acid which is core structure of betalain, (**b**) Amino acid which conjugates with betalamic acid to derive betaxanthin, (**c**) Yellow color giving betaxanthin, (**d**) Vulgaxanthin I which is a derivative of betaxanthin, (**e**) cyclo-*DOPA* which condenses with betalamic acid to give betacyanin, (**f**) Red-violet color giving betacyanin, and (**g**) Betanin which is a derivative of betacyanin [2,5,14,27,28,37,42].

Betalains are the main compounds associated with the displayed red color of flowers, fruits, and other plant tissues. Betalains are derivatives of tyrosine which are normally found in plant groups in the order of Caryophyllales including Amaranthaceae (*Beta vulgaris*), Cactaceae (*Opuntia*, *Pitaya*, *or Pitahaya*), Nyctaginaceae (*Bougainvillea*), Phytolaccaceae (*Phytolacca americana*), and Portulacaceae (*Portulaca grandiflora*) [2,3,27,37]. In Caryophyllaceae and Molluginaceae families, anthocyanin is more pronounced than betalain [2,28,32]. Their existence in high-class fungi, for example, agaric *Amanita muscaria*, *Hygrocybe*, and *Hygrophorus* have been reported as well [2,27,33,38]. Betalain compounds are accumulated in plant cell vacuoles, predominantly located in edible parts of plant tissues (epidermal and subepidermal), accompanied by other phytochemical compounds [4,5,6,37,39]. Betalains are nitrogen-containing heterocyclic compounds with high water affinity. Their exhibition of antioxidant activity is mainly based on their functional structure, for example, betalamic acid possesses conjugated double bonds that serve as reducing agents via electrons sharing [4]. Additionally, their ability to stabilize even after electron donation is responsible for its antioxidant property [4]. Depending on the type of groups (imino compounds or amino acid/derivatives) attached to the chromosphere of betalains, which is betalamic acid [4-(2-oxoethylidene)-1,2,3,4-tetrahydropyridene-2,6-dicarboxylic acid], two basic color compounds are derived [2,4,5,6,14,28,33,37,38]. Betaxanthin is obtained by conjugating betalamic acid with amines or amino acids, whereas betacyanin is derived from the condensation of *cyclo*-l-(3, 4-dihydroxylphenylalanine), known as *cyclo-DOPA,* or its glucosyl derivatives [2,3,4,5,6,14,27,28,33,37,38] (Figure 1). In the UV visible region, betaxanthin and betacyanin are detectable at the maximum wavelength absorption of 480 and 540 nm, respectively [27]. Betacyanin is responsible for the red color but also more tolerant to heat and stable to processing conditions than the yellow color giving betaxanthin [3,9,39]. 

Betaxanthin sources are limited, which makes for their low market demand despite their availability in the form of essential dietary amino acids, luckily, *Celosia* species are available as alternative sources of bright yellow color betaxanthin [40]. Indicaxanthin with prolin group (abundant in *Opuntia* species) and vulgaxanthin I (Figure 1d) with glutamine substituent, mostly found in *Beta vulgaris* species, are two prominent yellow color giving betaxanthins [4,27,28,37]. Their presence incites the fluorescence property of betaxanthin [4,38]. From the findings of Mikołajczyk-Bator and Pawlak [41], thermal degradation of red-violet pigments was three times lower than yellow color given compounds despite the antioxidant capacity of the latter, which tends to be higher after heat treatments.

Betanin, gomphrenin, amaranthin, and bougainvillein are four major types of betacyanin differed by the substituent groups attached to *cyclo-DOPA* moiety in ortho position (Figure 1g) [3,4,33,37,38]. Betanidin is the basic structural unit of most betacyanin derivatives, followed by, betanin (betanidin *5-O-ß*-glucoside) derives from glucosylation and acylation of aglycon betanidine (I) [3,27,33,37]. Betanin is the most stable red color compound, and its antioxidant ability is based on its donation of hydrogen and electron [4]. Presence of glucosyl substituent in C-6 hydroxyl group position and amine group in the ring system encourages radical scavenging activity of betacyanin [33,41], attributes of its anti-oxidative stress-related disorders, anti-cancer, and anti-inflammatory properties in specific plants and vegetables [33]. Phyllocathin (betanidin *5-O-ß*-malonyl-glucoside) and hylocerenin (betanidin *5-O-ß*-[3″-hydroxyl-3″-methyl-glutaryl] glucoside) are acylated forms of betacyanin [37]. In addition, isobetanin, betanidin, isobetanidin, prebetanin, neobetanin coexist in a small amount apart from betanin [4,34]. Those neo-derivatives are of interest due to their yellow color appearance derived from thermal degradation of *Beta vulgaris* and *Opuntia* species betanin [37]. Most degradation processes of betacyanin are isomerization, deglycosylation, dehydrogenation, hydrolysis, and decarboxylation [5,37,41].

Color variety of betalains is influenced by yellow color content to some extent [25], and their stabilities are determined by pH, light, heat, water activity, chelating agents, antioxidants, enzymatic reactions [4,37,43]. Peroxidases (POX), polyphenol oxidases (PPO), *ß*-glucosidase, and betalain oxidase are the most responsible enzymes for betalain degradation during processing [14,37]. The disturbance of some endogenous enzymes can be overcome by blanching at 70 °C for 2 min [13,27]. Betalain colorants are available in the form of concentrates [44] and dried powder through air, freeze, or spray drying [43,45], and are normally used in dairy and confectioneries and even in meat products [27,46], although addition in cold products is preferable because of their thermal sensitiveness. Furthermore, Sivakumar and coworkers [47] investigated their application for leather dying through the sonic extraction method. Coating of betalain pigments extracted from skin and pulp of *Cactus* fruit with acidified mucilage from the pulp of the fruit [48] or ionic gelation [49] can extend the shelf life as well as the stability of the pigments. Microencapsulation of beetroot pomace extracts and their applications in bakery products have also been explored recently [50]. 

### 2.2. Phenolic Compounds

Phenolic compounds (C_6_H_5_OH), also known as polyphenols, are aromatic metabolites made up of a polar functional (−OH) group bonded directly to an aromatic hydrocarbon ring [21]. Their ease of bonding to hydrogen raises their hydrogen bond formation ability for efficient water solubility in aqueous media [4]. Phenolic compounds are derivatives of aromatic amino acid phenylalanine ubiquitous in most plant kingdom with different glycosylated forms ranging from a simple phenolic molecule to high complex polymer molecules [21,51]. They are mostly localized in plant tissues (0.5–5 g in 100 g of dry weight) imparting pigments to their host [51]. As aromatic secondary metabolites, phenolics are anti-inflammatory, antimicrobial, and antithrombotic. They are willing to donate their electrons from the outermost shell to fulfil the leakage of free radicals and can extend the shelf-life of food products [16,17,21,52]. Consequently, phenoxyl radicals (PhO˙) are developed after transferring the hydrogen atom to free radical, which in turn can conjugate with adjacent hydroxyl or amine groups to enhance their stabilities, making them resistant to further oxidation process [4]. 

**Figure 2:**
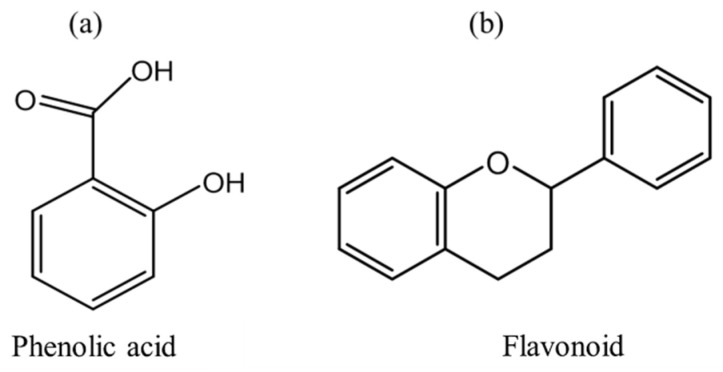
Basic structures of phenolic compounds: (**a**) Backbone structure of phenolic acid, and (**b**) Structure of flavonoid moiety [21,31].

Based on carbon atom numbers, phenolics, flavonoids, and tannins upon derivatization possess specific structures [21]. Among them, phenolic acids and flavonoids are the most active plant-based phenolic compounds (30% and 60% of total dietary polyphenols content) (Figure 2), they can easily be extracted with water, supercritical or subcritical water, and alcoholic solvents [17,53,54]. Benzoic acid derivatives (C_6_–C_1_) and cinnamic acid derivatives (C_6_–C_3_) are the two main classes of phenolic acids [21], whereas flavonols, flavones, flavanones, flavanols, isoflavones, flavanonols, and anthocyanidins are unique flavonoid compounds with variation in heterocyclic ring structures [17,21,31].

## 3. Generalities of Solid-Liquid Extraction

Classical extraction of target compounds from different types of matrix can be achieved through several cold or hot extraction methods: distillation; solvent extraction such as percolation, maceration, infusion, enfleurage; and cold compression [55]. Solvent selection should be suited with specific properties of compounds of interest. Apart from water, polar solvents such as alcoholic solvents are extensively used to upgrade extraction efficiency, although there are some oppositions of their application in the matter of total solid content increase [56]. Between 20%–50% *v*/*v* methanol or ethanol are the fundamentally used solvents for complete extraction of betalains. Being a non-toxic green solvent, extractions with ethanol solvent do not need any further purification step, while methanol is preferable for denaturation of enzymes and for overcoming the interference of water-soluble protein [14,27,33]. Biosynthesis reaction of compounds present in the matrix can be handled by acidifying the extraction medium; for example, enzymatic decolorization of betalain can be retarded by ascorbic acid, which interferes with oxidative activity of polyphenol oxidases or ß-gluconolactone and inhibits ß-glucosidase [33]. Moreover, ascorbic acid has some preservative effects on *Amaranthus* betacyanin, improved the stability of red color betalain, and upgraded yield of betanin to the highest as well [57]. However, as exogenous antioxidants, care should be taken in the application of ascorbic acids for antioxidant activity determination [14]. Chelating agents such as citric acid and EDTA (ethylene-diamine-tetra-acetic acid) are also prominent for acidification extraction of bioactive compounds in order to boost their yield [4]. In some cases, inorganic acid like hydrochloric acid is favorable to prevent the activity of endogenous enzymes [8,27].

## 4. Emerging Technology 

Handling of perishable vegetables is a big challenge for food processing industries and has led to the innovation of processing techniques to assure food quality and at the same time minimize food loss. Minimal processing is the preparation of food with minimal treatment and the smallest changes in food quality through modern processing technology. Attempts have been made to bring practical usage from emerging technologies to minimal food processing in the areas of tempering, vacuum drying, freeze-drying, dehydration, cooking, baking, roasting, pasteurization, sterilization, extraction, blanching, and direct microwave blanching [58]. Based on the characteristics of targeted products, thermal treatment methods (ultra-high temperature processing, aseptic or semi-aseptic heat treatment, sous-vid, infrared heating, high frequency or radiofrequency heating, ohmic heating, microwave heating, inductive electrical heating, etc.) and non-thermal treatment methods (low direct current electric fields, ionizing radiation, gamma irradiation, pulsed electric fields (PEF), UV light, pulsed light, laser light heating, ultrasonic wave heating, high-pressure processing, etc.) have been developed [59]. Currently used emerging technologies for the extraction of secondary plant metabolites (betalains and phenolic compounds) are microwave-assisted extraction (MAE), ultrasonic-assisted extraction (UAE), high pressure and temperature extraction (HPTE), supercritical fluids extraction (SFE), pressurized liquids extraction (PLE), pulse electric field extraction (PEF), gamma-irradiation-assisted extraction, and extraction by low direct current electrification [7,8,16,18,19,20,24,34,42,46,47,52,54,57,60,61,62,63,64,65,66,67,68,69,70,71,72,73,74,75,76,77,78,79,80,81,82,83,84,85,86,87,88,89,90].

### 4.1. Microwave Irradiation

**Figure 3:**
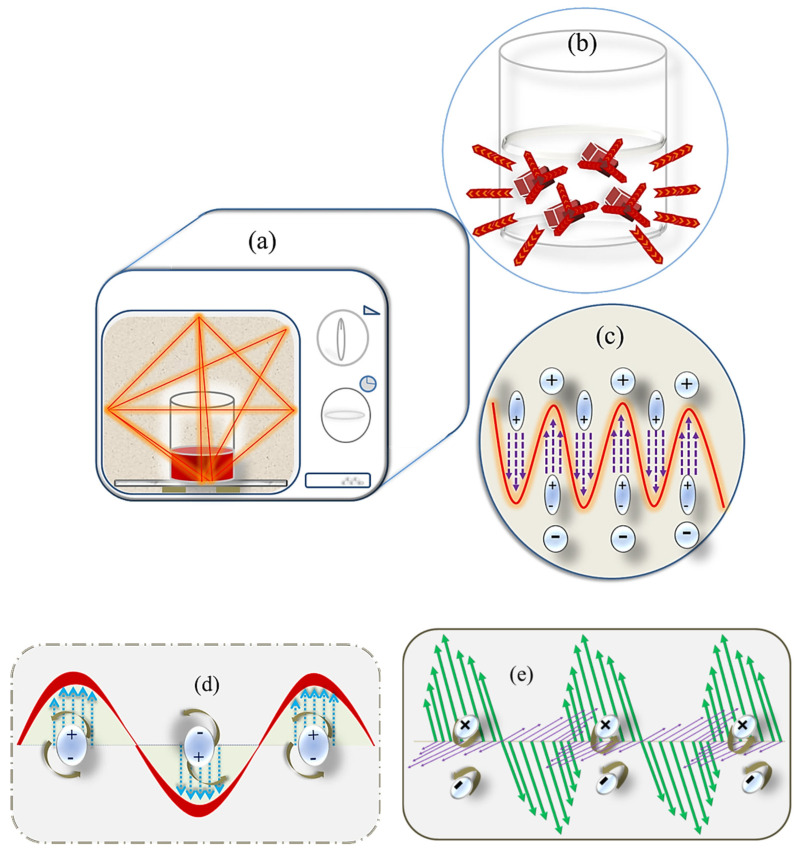
Treatment of microwave heat radiation for secondary plant metabolites extraction: (**a**) Domestic microwave oven heating system, (**b**) Heat diffusion from inside of the matrix to solvent medium and the surroundings, (**c**) Alignment of dipolar molecules and ions with electromagnetic wave, (**d**) Friction and rotation of dipolar molecules which heat up the matrix, and (**e**) Movements of ions with the electromagnetic wave.

In green chemistry, MAE is a well known novel technology with comparative advantages of reaction time reduction and lower or no solvent requirement over conventional extraction techniques [91]. Electromagnetic waves with frequencies between 300 MHz and 300 GHz are noted as microwave and previously applied for navigation and telecommunication operations [53,92]. Based on their application purposes, two frequency ranges are classified as 2450 MHz for home usage with adjustable power output <1000 W and 915 MHz for the industrial one [23,53,55,91]. Being a segment of the electromagnetic spectrum, like visible light, the phenomenon of bending, reflection, refraction, and absorption of microwave radiation by the medium through which it passes are unavoidable [92]. Likewise, the degree of absorptivity or transmissivity of transmitted waves relies on the electromagnetic properties of the treated object, for instance, dielectric property, polarity, permittivity, and the shape of the object. Another important factor is in situ water content of the matrix as the level of swelling and rupture of the matrix can be varied with it [93]. The contradiction of electromagnetic wave is based on its electric and magnetic wave propagation oscillating in a perpendicular direction to each other with wavelength (1 m~1 mm) [23,53,55,91]. Herein, the electric part if only absorbed by natural biological materials [94] consequently leads to heating process occurring in the medium with absorption property of microwave [55,91]. Microwave radiation is non-ionized with photon energy ranging from 3.78 × 10^−6^ eV to 1.01 × 10^−5^ eV, and interaction process occurs by heat conduction mode [55]. Moreover, the transformation phenomenon of kinetic to thermal energy is mainly concerned with the polarization potential of the polar molecules and their surroundings [23,92]. The protocol is that once polar molecules present in a substance are hit by the electromagnetic beams, they become energetic and swing with the alternative movement of electric field, and consequently the alternative action of alignment and realignment of polar molecules creates friction between them, which in turn lead to the heating up of the surroundings [53,92] (Figure 3). Meanwhile, ionic components which are present in the substance migrate according to the electric field [91] (Figure 3). This intermolecular friction and ionic movements occur several million times (4.9 × 10^9^ times at a frequency of 2450 MHz) per second and raise the internal pressure of cell [91]. Consequently, higher internal pressure encountered due to rapid vaporization of in situ water could rupture the cell wall and enclosed substances inside the cell wall could be forced out of the cell at a high rate [23]. 

### 4.2. Mathematical Terms

Electric loss tangent (tan δ) of materials, which is also known as dissipation factor, quantifies the transformation of electric and magnetic energy to thermal energy in the materials; it can be explained in terms of dielectric loss (ε″) and dielectric loss constant (ε′) as given below in (Equation (1)) [23,91,92,94]:

(1) $$D_{f}=\tan\delta =\frac{\varepsilon^{\prime \prime }}{\varepsilon^{\prime }}$$

Absorption, transmission, and reflection abilities of different materials, known as electric permittivity, can be tracked in several ways. The absolute permittivity elucidates only how the material interacts with applied electromagnetic waves for nonionizing radiation and nonmagnetic materials [94]. Alternatively, it can be said that nonmagnetic materials rely on dielectric permittivity (ε*) to interact with electromagnetic waves and can be expressed by dielectric constant (ε′) and dielectric loss (ε″) shown in (Equation (2)); [94]:

(2) $$\varepsilon^{*}\;=\;\varepsilon_{r}^{\prime }-j\varepsilon_{r}^{\prime \prime }$$

Real and imaginary parts of permittivity represent dipolar oscillation and damping [92], and are valued depending on the motion of dipoles, nature of materials, high or low frequency, elevated or reduced temperature, and also the concentration of aqueous ionic solution [94]. Commonly used solvents with their loss tangent values are listed in Table 2. Relative permittivity (ε) can be estimated from electric displacement field of material (*D*, C · m^−2^) which is divided by vacuum (*D_vacuum_*, C · m^−2^) [92]. In dielectric material, conversion of electrical energy into thermal energy is defined by its loss factor. Consequently, dipole rotation and electrical conduction are assumed as loss factors in microwave heating, (Equation (3)):

(3) $$\varepsilon_{r}^{\prime \prime }\;=\;\varepsilon_{d}^{\prime \prime }+\varepsilon_{\sigma }^{\prime \prime }$$

where ε_d_″ is relative dipole loss and ε_σ_″ is relative ionic loss.

If the components are homogeneous, their shape is more responsible for the effective permittivity of the mixtures, especially when the particle size is smaller than the wavelength [94]. Literally, ordinary foods possess the penetration depth of electromagnetic waves (10–15 mm) [94]. The penetration level of microwave through the substance can be traced by the degree of reflection, transmission, and absorption of the wave by the host substance; for example, pure water has a low degree of reflection and transmission but a high degree of absorption of the wave [92]. Dissipated microwave power (*P_D_*, W) in a material can be estimated from electric field strength (*E*, V · m^−1^), and frequency (*f*, Hz) as reported by [92,94] (Equation (4)):

(4) $$P_{D}=k\;\cdot E^{2}\cdot f\cdot \varepsilon^{\prime \prime }$$

where *k* is a constant value of 55.61 × 10^−14^ C · m^2^ · V^−1^. Due to direct proportionality of dissipated microwave power (P_D_) to the absorptivity of the material, the attenuation of microwave differs according to the depth of penetration (z) and can be estimated as expressed in Equation (5):

(5) $$z=\frac{\lambda }{2\pi }\cdot \sqrt{\frac{2}{\varepsilon^{\prime }\left(\sqrt{1+tan^{2}\delta }-1\right)}}$$

Frequency of applied radiation (f, Hz) and speed of the wave (c, m · s^−1^) can be used to evaluate wavelength (λ, m) [92].

### 4.3. Microwave-Assisted Extraction (MAE) of Betalains and Phenolic Compounds 

Microwave irradiation is proposed as an effective alternative way of recovering bioactive compounds from agro-industrial wastes with superior benefits such as high reproducibility within short treatment period, minimal solvent and energy consumption, and simple manipulation as opposed to classical solid–liquid-based extraction methods (soxhlet, rotary, maceration, and heat refluxing), supercritical water extraction, and even ultrasonic extraction [16,18,20,52,54,60,61,62,63,64]. In advance, MAE has brought 1.7 times greater yield percent of phenolic [65], whilst operation time was two times shorter than UAE [54]. Alongside, 5 min of microwave irradiation could bring an equivalent amount of phenolic and flavonoid compounds with an hour of ultrasonic treatment time [61]. However, UAE was pointed out as the better choice in the investigations of [22,63], in this case, the discrepancy in applied frequency and power wattage should be taken into account. Somehow, it is undeniable that both MAE and UAE are better in internal or external mass transfer compared to classical methods. In UAE, the formation of tiny gas bubbles in the solvent medium by mechanical wave encourages several micro-injections into the cell wall, which improves the solvent transport. Herein, sonication of water can propagate highly reactive hydroxyl radicals which may have some effect on the extraction of targeted products [52,66].

**Figure 4:**
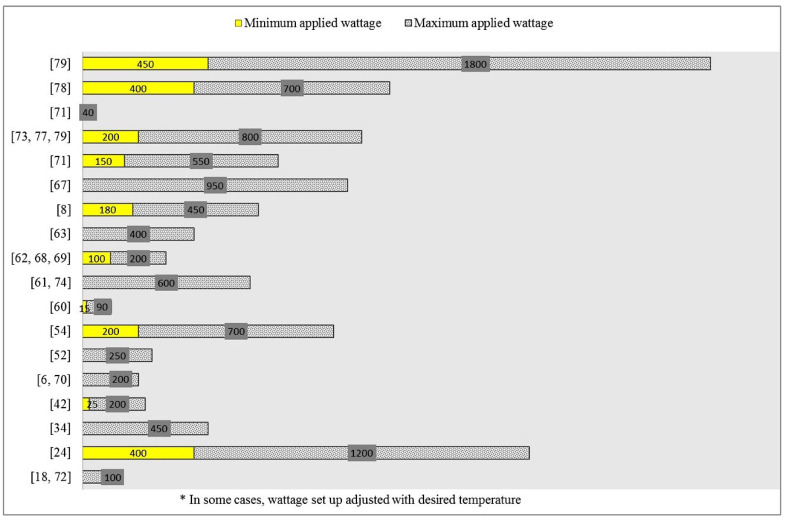
Ranges of power output for extraction of secondary plant metabolites (betalains and phenolic compounds).

**Figure 5:**
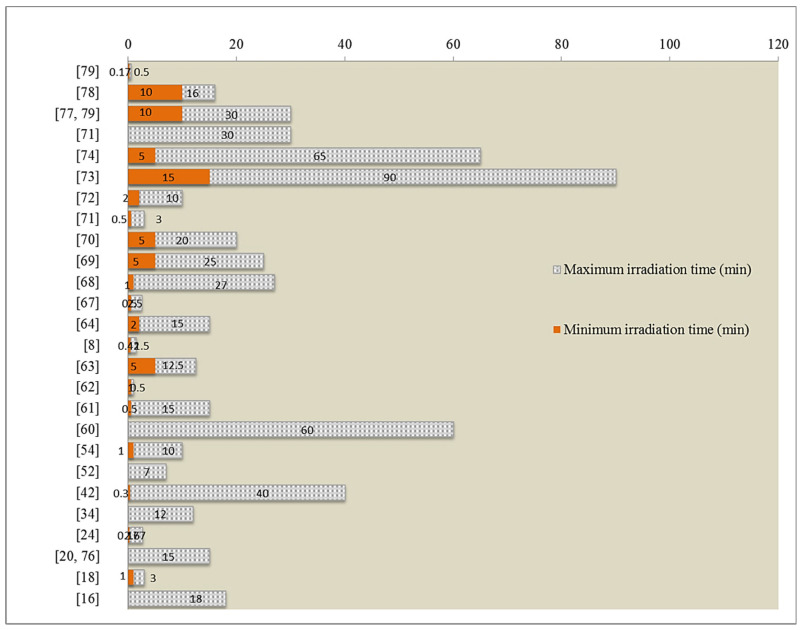
Ranges of microwave irradiation time (min) for extraction of phytochemicals (betalains and phenolic compounds).

Heating mode, duty cycle, power or heat energy, and temperature in an advanced microwave set up are basic operational parametric modes in MAE of betalains and phenolic compounds. Response surface methodology (RSM) is a suitable tool to approach the efficiency of MAE through the interaction of process variables despite the fact that the predicted values estimated by the model are only within the experimental limit [9,18,62,63,67,68,69,70,71,72,73]. Central composite design was mostly chosen for the investigation of each or parallel effects of process variables on the corresponding response. Based on the response level whether high or low, the weight of the factor on the response could be judged. Here again, the levels of the factors optimized by RSM support to be in between, i.e., neither at most nor lowest level or else the performed limits are regarded as insufficient for the design to fully predict the optimum condition of the variables within those limits [26]. For example, it can be said that the model well predicted the optimum condition of the extraction process if the highest amount of total phenolic compounds (39 mg GAE/gDW) was achieved at ethanol concentration (31.33%), solvent to material ratio (32.21 mL/g), temperature (52.24 °C) among the variables which were ethanol concentration (20–40%), solvent to material ratio (20–40 mL/g), temperature (40–60 °C) [73]. Table 3 and Table 4 represent some reported optimum conditions, within experimental variable ranges, for the extraction of betalains and phenolic compounds from fruit and vegetable as well as their wastes. In advance, some example of power output and time setting to accomplish MAE of secondary plant metabolites are demonstrated in Figure 4 and Figure 5.

In MAE, both the solvent and the sample undergo transformation by electromagnetic energy of high value based on ionic conduction and dipole rotation (either permanent or induced by the electric field). Ionic conduction, which is due to the electrophoretic migration of dissolved ions, increases solvent penetration into the matrix and facilitates the solvation of targeted compounds [91]. Influence of solvent type on microwave extraction of desired phenolic compound quantity has been investigated with pure water, pure and aqueous forms of acetone, ethanol and methanol, and of them, 50% (*v*/*v*) ethanol was observed to be most effective [61,64,78,80]. Presence of a suitable amount of water in the solvent somehow can improve the swelling of the plant matrix, increasing the contact surface area of the matrix with the solvent [62]. However, it is controversial whether inert solvent to microwave radiation is better than the absorbed one or not as acetone performed better in phenolic compounds extraction possibly due to transparent property to microwave radiation subsequently allowing plant cells receive direct contact of the radiation [19]. Moreover, it should be noted that microwave can rapidly lose its energy before it reaches the innermost part of the medium if the medium it passed through is in high absorptivity of radiation [92]. 

Applied power output for microwave irradiation influences phenolic compounds [73], and it is obvious if irradiation time is short [59], which possibly leads to accelerated diffusion of the solvent into the matrix and in turn leaching out of the materials [23]. Prolonged heating via elevated temperature treatments can jeopardize pigment retentions; in this case, the acidification method can be considered for decompartmentalization of color compounds after microwave treatment [24]. Synergistic effects of processing time and temperature or microwave power, solvent concentration, and matrix-to-solvent ratio on mass transfer rate differ for specific secondary plant compounds, thus process condition adjustment should be focused on targeted compounds in order to distribute radiations uniformly [17,24,73]. Reported SEM (scanning electron microscopy) results showed that cell wall thinning and holes formation were prominent in microwave treatment rather than traditional heating modes [65,80,82,95,96]. This is because microwave penetrates the matrix and denaturalizes the cell wall in which bioactive compounds are trapped in a short time, subsequently letting them diffuse quickly into the solvent medium. However, the superheating effect can be encountered as a consequence, which is more pronounced with pure water solvent due to its quite low dissipation factor but high dielectric constant, facilitating the absorption of the microwave [92]. Since microwave heating is based on the alignment of the polar molecules back and forth, the transformation of kinetic energy to thermal energy is more intensive when their movement cannot catch up the frequency of applied microwave, which is the so-called dumping process [62]. That is why intermittent modes of microwave irradiation and cooling in between extraction steps were found to be effective [21,24,52,61,64,81].

## 5. Other Applications 

Apart from betalain and phenolic compounds, MAE has been widely used in the extraction of pectin with some improvements in quantity and quality, degree of esterification, and gel strength [97]. Moreover, essential oil extractions from different sources via advanced microwave-assisted techniques have been realized [93,98,99]. The combination of novel extraction methods which are microwave, ultrasonic wave, enzymatic, and also alkali assisted extraction have been complimented as well [90,95,100,101] (Table 5). Other improved MAE methods are in situ extraction of essential oils with microwave oven [102], MAE of multi-elements from food materials via perchloric acid and hydrogen peroxide [103], MAE and GC-FID quantification of total branched-chain fatty acids in lamb subcutaneous adipose tissue [104]. Additionally, some other modified techniques of microwave irradiation such as vacuum microwave-assisted extraction, nitrogen-protected microwave-assisted extraction, dynamic microwave-assisted extraction, pressurized solvent free microwave-assisted extraction, radiation-assisted hydro-distillation, vacuum microwave hydro-distillation, microwave-assisted steam distillation, vacuum microwave-assisted hydro-gravity, microwave dry-diffusion and gravity have been mentioned in the reviews of [23,91]. Apart from extraction, microwave application has been extended to processing of beetroots which has more effectiveness in oxidative stability of mayonnaise than roasted or boiled ones, thereby preserving betalains and phenolic compounds [105].

## 6. Conclusions

As natural bioactive compounds rich in antioxidants, therapeutic properties of betalain and phenolic compounds are numerous. Although they are sensitive to heat, pH, light, etc., not only during processing but also throughout the storage, their presence in food wastes and residues even after processing is noteworthy as well. Currently, their practical usages in food industries, fortification, and supplementation of foods, cosmetics, and pharmaceuticals are thriving. Therefore, further thorough investigations for extraction of these valuable compounds via modern technologies, meantime maintaining their original properties as much as possible, are still needed to be explored.

Most of MAE discussed in this review are operated in batch, although semi-continuous or continuous modes are also available. Since microwave irradiation encourages the direct interaction of vast electromagnetic waves and the target material, higher power ensures enough contact of the microwave with the matrix to expedite the isolation of the compounds trapped in the cell wall. Therefore, high temperature or power and short irradiation time is a beneficial combination for MAE of plant materials. Higher extraction time is not necessary once after the equilibrium state between the solvent and the matrix is reached since it can lead to the degradation of most bioactive compounds. All in all, if it is in proper use, microwave can be the best option for bioactive compounds extraction for the sake of simplicity as it can easily be set up or run by the home used microwave oven, high efficiency as the heating is built at the innermost part of the matrix and diffuses to the surrounding, saving energy as the operation can be completed in few minutes, and cleanliness as it is the only way of solvent free or lowest solvent used extraction.

## Figures and Tables

**Table 1:** Natural colors applied in the food and cosmetic industries, their E numbers, and common sources [3,25,28,35,36].

Group	Compound/Product	Colour	Code	Common Sources
-	Curcumin, turmeric oleorisin	Yellow-orange	E100	Turmeric
-	Riboflavin (vitamin B2), riboflavin-5′-phosphate	Yellow-orange	E101(i), E101(ii)	Eggs, green vegetables, milk and other dairy products, meat, mushroom, almond
-	Riboflavin-5-sodium phosphate	Yellow	E106	Eggs, organ meats (kidney and liver), lean meat, milk, green vegetables
-	Carminic acid	Magenta-red/ crimson	E120	Cochineal insect
-	Chlorophyll	Green	E140, E141	Grass, alfalfa leaf, *Tagetes erecta* (marigold flowers), spinach
-	Caramel	Brown	E150	Modified sugar
-	Vegetable carbon	Black	E153	Vegetables
Carotenoid	α-carotene, β-carotene, γ-carotene	Orange-red, red, yellow, amber, brown	E160a	Carrot, pumpkin, apricot, sweet potato, beans, spinach, kale, collard greens, papaya, bell peppers, tomatoes, green leafy vegetables
Carotenoid	Annatto	Orange-red	E160b	Fruit of the achiote tree
Carotenoid	Paprika oleoresin	Red	E160c	*Capsicum annuum or Capsicum frutescens*
Carotenoid	Lycopene	Bright to deep red	E160d	*Solanum lycopersicum* (tomatoes), watermelon
Carotenoid	β-Apo-8′-carotenal	Orange to orange-red	E160e	Spinach, citrus fruits
Carotenoid	Lutein	Orange-red to yellow	E161b	Green leafy vegetables and fruits, yellowish flowers
Carotenoid	Canthaxanthin	Violet	E161g	Mushroom, crustaceans, fish, eggs
Nitrogen containing compound	Betalains/ betanin	Red violet to yellow (pH < 3 (more reddish), pH > 7 (more yellowish))	E162	Mangosteen, beetroot, dragonfruit, red cabbage, swiss chard, *Opuntia*
Phenolic	Anthocyanin	Dark purple (pH 1 (red), pH 4–5 (colorless), pH < 7 (purple), pH < 8 (deep blue), pH < 12 (yellow/brown))	E163	Black currant, berries, cherry, plum, grape, red cabbage, *Opuntia*, hibiscus, rose, onion
Carotenoid	Saffron	Yellow-orange-red	E164	*Crocus sativus*

**Table 2:** Reported mathematical parametric values associated with commonly used solvents for phytochemical recovery through MAE (Microwave-Assisted Extraction).

Solvent	Loss Tangent	Dielectric Constant (ε′)	Dielectric Loss (ε″)	Reference
water	0.123	80.4	9.8892	[23,53,58]
ethanol	0.941	25.7	24.1837	[23,53]
methanol	0.659	32.7	21.5493	[23,53]
acetone	0.054	20.6	1.1124	[53]

**Table 3:** Recovery of betalain color compounds through microwave irradiation.

Raw Material	Solvent	Product	Process Optimum Conditions			Reference
			Results	Time	Power	
Red beetroot peel	acidified water	betanin	229.26 mg/L (predicted by RSM)	0.95 min	224.61 W	[8]
	ethanol		472.11 mg/L (predicted by RSM)	1.25 min	384.25 W	
Red beetroot	pure water	betaxanthin	1.25 mg/g of freeze dried red beet	2.2 min/ 1.7 min	400 W, 100% nominal	[24]
	acidified water	betacyanin	1.88 mg/g of freeze dried red beet			
Red beetroot	pure water	betacyanin	52.2 mg/g of fresh matter	3 min x 4 times	450 W	[34]
		betaxanthin	42.8 mg/g of fresh matter			
*Opuntia* fruit peel	34.6% methanol	betalain	201.6 mg/g of extract	2.5 min	400 W	[63]
Dragon fruit peel	pure water	betalain	9 mg/L (predicted by RSM)	8 min	100 W	[72]
White-fleshed red pitaya peel	pure water	betacyanin	1.66 mg/g of dry extract	5 min	600 W	[74]

RSM = response surface methodology.

**Table 4:** Total phenolic compound (TPC) recovery by microwave radiation treatment.

Raw Materials	Extraction	Analytical Method	Process Optimum Conditions			Reference
			Results	Time	Power	
*Coriandrum sativum* (spice)	50% ethanol	FC	0.82 mg GAE/g of DW	18 min	200 W, 50% nominal	[16]
*Cinnamomum zeylanicum* (spice)			16.8 mg GAE/g of DW			
*Cuminum cyminum* (spice)			11.6 mg GAE/g of DW			
*Crocus sativus* (spice)			29.4 mg GAE/g of DW			
*Lessonia trabeculate* (Brown algae)	70% methanol	FC	0.74 mg GAE/g of DW	15 min	intermittent	[20]
*Lessonia nigrecens* (Brown algae)			1.07 mg GAE/g of DW			
*Ascophyllum nodosum* (Brown algae)			1.4 mg GAE/g of DW			
*Laminaria japonica* (Brown algae)			0.73 mg GAE/g of DW			
Rosemary	96% ethanol	FC	0.9 mg GAE/g of fresh leaf	7 min	250 W (intermittent)	[52]
	pure water		0.1 mg GAE/g of fresh leaf			
Grape seed	methanol	FC	67.88 mg GAE/g of DW	60 min	60 W	[60]
Grape skin			7.33 mg GAE/g of DW			
Eucalyptus leaf	50% ethanol	FC	76.6 mg GAE/g of fresh leaf	5 min	600 W (intermittent)	[61]
Olive leaf	50% ethanol	FC	88.3 mg TAE/g of DW	15 min	intermittent	[64]
Peanut skin	30% ethanol	FC	143.6 mg GAE/g of skins (predicted by RSM)	0.5 min	950 W, 90% nominal	[67]
Broccoli	72.06% methanol	FC	21.39 mg GAE/g of DW	16.94 min	159.33 W	[68]
Wine lee	75% ethanol (acidified)	FC	36.4 mg GAE/g of lee extract powder	17 min	200 W	[69]
(*Ipomoea Batatas*) Sweet potato leaf	53% ethanol	FC	61.26 mg GAE/g DW	2.05 min	302 W	[71]
*Melastoma sanguineum* Fruit	31.33% ethanol	FC	39.02 mg GAE/g of DW	45 min	500 W	[73]
*Lycium spp.* leaf	pure methanol	FC	6.65 mg GAE/g of DW	30 min	40 W	[75]
Buckwheat	50% ethanol	FC	18.5 mg GAE/g of buckwheat	15 min	-	[76]
Pitaya peel	pure water	FC	5.8 mg GAE/g of extract	20 min	400 W	[77]
Sour cherry pomace	50% ethanol	FC	14.14 mg GAE/g of DW	12 min	700 W	[78]

GAE = gallic acid equivalent, FC = folin-ciocalteu analytical method, TAE = tannic acid equivalent, DW = dry weight.

**Table 5:** Some other applications of advanced MAE and their findings.

Sources	Methods	Advantages	References
Sorghum husks	Ultrasonic-microwave assisted extraction of colorant (UMAE)	Higher in thermal stabilities and yield percent (3.6 times) with high contents of apigeninidin and luteolinidin than conventional shaking	[90]
Aromatic herbs	Enhanced solvent free microwave-assisted extraction (ESFMAE)	ESFMAE increased in oxygenated compound content which was more odoriferous than monoterpene hydrocarbons	[93]
Cherry seeds	Ultrasonic-microwave-assisted aqueous enzymatic extraction (UMAAEE)	Compared to Soxhlet extraction, oil by UMAAEE possessed superior physicochemical properties and higher content of bioactive constituents	[95]
Tunisian cumin (*Cuminum cyminum* L.) seeds	Microwave hydrodiffusion and gravity extraction (MHGE)	MHGE successfully improved the EO yield with high amount of oxygenated compounds in shorter extraction time, less electrical consumption, lower carbon dioxide emissions, and smaller volume of waste water	[96]
Rosemary plants	Microwave hydro-distillation (MHD)	MHD was superior in terms of saving energy and extraction time compared to hydro-distillation	[98]
*Foeniculum vulgare* Mill. seeds	Enhanced solvent free microwave-assisted extraction using double walled reactor (ESFMAE)	ESFMAE method was faster, cleaner, less cost and energy usage, and better essential oil composition than hydro-distillation method	[99]
Arabica coffee beans	Ultrasonic-microwave assisted extraction of green coffee oil (UMAE)	Extraction yields of two diterpenes (cafestol and kahweol) by UMAE were significantly higher than that of solvent method	[100]
Corn brans	Ultrasonic-microwave assisted alkali extraction of arabinoxylan (UMAAE)	By UMAAE, water-unextractable arabinoxylan (WUAX) showed good DPPH radical scavenging activity and strong Fe^2^+ chelating activity	[101]
*Schisandra chinensis* Baill fruits	Ionic liquid-based microwave-assisted extraction (ILMAE)	ILMAE method shortened the energy consumption time, improved the extraction efficiency of lignans as to reflux extraction	[106]
Flowers of *Ulex europaeus* L.	Microwave hydrodiffusion and gravity extraction (MHGE)	MHGE allowed an efficient water removal from the material, and could be suitable for extraction of antioxidant rich aromatic compounds	[107]
Tomatoes	Deep eutectic solvent-based microwave-assisted dispersive liquid–liquid microextraction preconcentration of multiclass pesticide residues in tomato samples (DES-MWA–DLLME)	DES-MWA–DLLME represented good repeatability, high (enrichment factors) EFs, low (limit of detection) LODs	[108]
